
JOURNAL INFORMATION
==============================
NLM Title Abbreviation: ScientificWorldJournal
Journal ID: ScientificWorldJournal
NLM Journal ID: 101131163
Journal ID: TSWJ

The Scientific World Journal

ISSN: 2356-6140
EISSN: 1537-744X
Publisher: Wiley

ARTICLE INFORMATION
==============================
PMCID: PMC5917169
PMID: 16604256
DOI: 10.1100/tsw.2006.89
Article ID: 717819
Article version: 1
Subjects: Directions in Science

Common Neuroprotective Effects of Endothelin Receptor A Blockade and Erythropoietin During Hypoxia-Associated Cell Injury. Perspectives of Combined Treatment

Danielyan Lusine 1 *
Buniatian Gayane H. 2
Gleiter Christoph H. 1
1 Department of Clinical Pharmacology, University Hospital of Tuebingen, Otfried-Mueller Str. 45, D-72076 Tuebingen, Germany
2 Institute of Biochemistry, Medical Faculty of University of Leipzig, Liebig Str. 16, D-04103, Leipzig, Germany
*Lusine Danielyan: lusine.danielyan@med.uni-tuebingen.deAcademic Editor: Martin Goette

Publication date: 2006
Electronic publication date: 2006 Apr 5
Volume: 6
First page: 449
Last page: 451
Received 2006 Feb 27; Revised 2006 Mar 27; Accepted 2006 Apr 5
Copyright: Copyright © 2006 Lusine Danielyan et al.
Copyright year: 2006
License: This is an open access article distributed under the Creative Commons Attribution License, which permits unrestricted use, distribution, and reproduction in any medium, provided the original work is properly cited.
License URL: https://creativecommons.org/licenses/by/3.0/

Keywords: ischemia, stroke, Alzheimer's disease, neurodegeneration, apoptosis, neurons, astrocytes, proliferation, differentiation, endothelin receptor A antagonist, endothelin receptor B antagonist, BQ-123, BQ-788, erythropoietin

==============================
Directions in Science
*TheScientificWorldJOURNAL* (2006) 6, 449–451
ISSN 1537-744X; DOI 10.1100/tsw.2006.89

TheScientificWorldJOURNAL
www.thescientificworld.com

### Common Neuroprotective Effects of Endothelin Receptor A Blockade and Erythropoietin During Hypoxia-Associated Cell Injury. Perspectives of Combined Treatment

Lusine Danielyan[1,]*, Gayane H. Buniatian[2], and Christoph H. Gleiter[1]

*[1]Department of Clinical Pharmacology, University Hospital of Tuebingen, Otfried-Mueller Str. 45, D-72076 Tuebingen, Germany; [2]Institute of Biochemistry, Medical Faculty of University of Leipzig, Liebig Str. 16, D-04103, Leipzig, Germany*

**E-mail**: lusine.danielyan@med.uni-tuebingen.de

*Received February 27, 2006; Accepted March 27, 2006; Published April 5, 2006*

---

**KEYWORDS**: ischemia, stroke, Alzheimer's disease, neurodegeneration, apoptosis, neurons, astrocytes, proliferation, differentiation, endothelin receptor A antagonist, endothelin receptor B antagonist, BQ-123, BQ-788, erythropoietin

---

In the last decade, numerous findings opened new perspectives for neuroprotection in the treatment of acute and chronic neurodegenerative diseases such as stroke, neurotrauma, and Alzheimer's disease. The concept of neuroprotection includes a multitude of processes leading to the protection of cells in the central nervous system (CNS) against degeneration and death or/and compensation of the functional loss by generation of new functionally active cells.

Over more than 10 years of investigations of the local endothelin (ET) system in the CNS have shown a stong involvment of this potent vasoconstrictor in the pathogenesis of ischemic brain injury. ET-1 was shown to be markedly up-regulated after ischemic brain injury[1,2]. However, the literature data concerning the effects of ET-1 during ischemic damage are contradictory: Some of them show that the exogenous application of ET-1 can lead to an exacerbation of ischemic damage[3,4] and that the blockade of ETA/B-receptors (ETA/B-R) reduces the focal cerebral ischemic injury[5]. In contrast, Ho et al.[6] demonstrated that ET-1 protects against ischemic injury and that up-regulation of astrocytic ET-1 expression is essential for the survival of astrocytes. ET-1-deprived astrocytes were more vulnerable to hypoxia and ischemia. Both ET receptor types (ETA- and ETB-R) appear to play an important role in the functions of different types of cells, CNS cells inclusively. ET-R subtypes can regulate the control of glutamate clearance[7], dopaminergic activity[8], and cell survival during different types of injury[9,12].

During degenerative diseases of the brain, accompanied by decreased oxygen supply, disturbances of brain-specific homeostasis, and cell death, the renewal of cell populations may provide a balance between the functionally active and apoptotic cells. Despite numerous studies demonstrating the critical role of astrocytic apoptosis in the pathogenesis of acute and chronic disorders such as cerebral ischemia or Alzheimer's disease (for review see [10,11]), the mechanisms regulating the astrocytic survival and their capacity to counteract pathological situations are not well understood.

*Corresponding author.
©2006 with author.
Published by TheScientificWorld, Ltd.; www.thescientificworld.com

Therefore, in our recent study, we investigated the role of ETA and ETB receptors in the protective mechanisms underlying the resistance of astroglial cells to hypoxia[12]. It was also of high interest to compare the protective capacities of ETA-R blockade with those of erythropoietin, a cytokine with established neuroprotective effects. The *in vitro* investigations performed on astroglial primary cultures under normoxic and hypoxic conditions have shown that the administration of an ETA-R antagonist, BQ-123, led to an increase in cell number and inhibited the hypoxia-induced apoptosis and cytotoxicity. These effects of BQ-123 were abolished in cultures simultaneously treated with the ETB-R antagonist BQ-788. Further on, the rejuvenation of astroglial cells after administration of BQ-123 under hypoxic and normoxic conditions was shown by an increased number of nestin- and glial fibrillary acidic protein (GFAP)-positive astrocytes accompanied by decrease of cells solely expressing GFAP. The protection provided by BQ-123 to astroglial cells against hypoxia-induced cytotoxicity as well as its cell proliferation-inducing effect was similar to that of erythropoietin (EPO). The antiapoptotic effects of BQ-123 on astroglial cells could be enhanced by simultaneous application of EPO. A significant increase in the number of nestin+/GFAP- under treatment with EPO or BQ-123 manifested the slowing down of astrocyte differentiation[12]. In our second study, we demonstrated for the first time that the protective effects of BQ-123 are associated not only with its antiapoptotic and rejuvenating activities on astrocytes, but also with the ability to ameliorate the resistance of neurons and neuronal progenitors to decreased oxygen supply[13]. The BQ-123-influenced enlargement of the population of differentiated neurons and undifferentiated neuronal precursor cells shown in this study points at an activation of mechanisms supporting the generation of neurons as well as an increased survival of the cells. The number of undifferentiated neurons and the survival of differentiated neurons under normoxic and hypoxic conditions was increased under treatment with EPO either. This effect of EPO on proliferation of pre-existing neuronal precursors and enhancement of their differentiation into the cells of neuronal lineage was shown also in other studies[14,15]. Similar neuroprotective potential provided by the blockade of ETA-R and treatment with EPO hints at a possible implementation of these compounds in the treatment of hypoxia-associated disorders of the brain.

Taken together, the findings mentioned above challenged the exclusivity of vasoactive effects of ETA-R blockade, showing its strong neuroprotective capacity. The antiapoptotic, survival-enhancing, and rejuvenating effects of ETA-R blockade and EPO alone, and their additive effects on neurons and astrocytes, can be used in the treatment of the wide spectrum of acute and chronic neurodegenerative diseases such as stroke, neurotrauma, and Alzheimer’s disease. However, in order to provide the “proof of the concept” for the clinical implementation of ETA-R blockade alone and in combination with EPO, further attempts should be done to explore neuroregenerating characteristics of ETA-R blockade and EPO in *in vitro* and in vivo models of different types of neurodegeneration.

### REFERENCES

1. Bian, L.G., Zhang, T.X., Zhao, W.G., Shen, J.K., and Yang, G.Y. (1994) Increased endothelin-1 in the rabbit model of middle cerebral artery occlusion. *Neurosci. Lett.* **174**, 47–50.
2. Wilette, R.N., Ohlstein, E.H., Pullen, M., Sauermelch, C.F., Cohen, A., and Nambi, P. (1993) Transient forebrain ischemia alters acutely endothelin receptor density and immunoreactivity in gerbil brain. *Life Sci*. **52**, 35–40.
3. Agnati, L.F., Zoli, M., Benfenati, F., Bagini, G., Zini, I., Hallstrom, A., Ungerstedt, U., Toffano, G., and Fuxe, K. (1991) A new model of focal brain ischemia based on the intracerebral injection of endothelin-1. *Ital. J. Neurol. Sci*. **12**, 49–53.
4. Gartshore, G., Patterson, J., and Macrae, I.M. (1997) Influence of ischemia and reperfusion on the course of brain tissue swelling and blood-brain barrier permeability in a rodent model of transient focal cerebral ischemia. *Exp. Neurol*. **147**, 353–360.
5. Barone, F.C., White, R.F., Elliott, J.D., Feuerstein, G.Z., and Ohlstein, E.H. (1995) The endothelin receptor antagonist SB 217242 reduces cerebral focal ischemic brain injury. *J. Cardiovasc. Pharmacol*. **26**, S404–407.
6. Ho, M.C., Lo, A.C., Kurihara, H., Yu, A.C., Chung, S.S., and Chung S.K. (2001) Endothelin-1 protects astrocytes from hypoxic/ischemic injury. *FASEB J*. **15**, 618–626.
7. Matsuura, S., Ikegaya, Y., Yamada, M.K., Nishiyama, N., and Matsuki, N. (2002) Endothelin downregulates the glutamate transporter GLAST in cAMP-differentiated astrocytes in vitro. *Glia* **37**, 178–182.

8. Kohzuma, M., Kataoka, Y., Koizumi, S., Shibaguchi, H., Nakashima, M.N., Yamashita, K., Niwa, M., and Taniyama, K. (1994) ETB receptor involvement in stimulatory and neurotoxic action of endothelin on dopamine neurones. *Neuroreport* **5**, 2653–2656.
9. Yagami, T., Ueda, K., Asakura, K., Kuroda, T., Hata, S., Sakaeda, T., Kambayashi, Y., and Fujimoto, M. (2002) Effects of endothelin B receptor agonists on amyloid beta protein (25-35)-induced neuronal cell death. *Brain Res*. **948**, 72–81.
10. Takuma, K., Baba, A., and Matsuda, T. (2004) Astrocyte apoptosis: implications for neuroprotection. *Prog. Neurobiol*. **72**, 111–127.
11. Nakase, T., Sohl, G., Theis, M., Willecke, K., and Naus, C.C. (2004) Increased apoptosis and inflammation after focal brain ischemia in mice lacking connexin43 in astrocytes. *Am. J. Pathol*. **164**, 2067–2075.
12. Danielyan, L., Gembizki, O., Proksch, B., Weinmann, M., Morgalla, M., Wiesinger, H., Buniatian, G.H., and Gleiter, C.H. (2005) The blockade of endothelin A receptor protects astrocytes against hypoxic injury: common effects of BQ-123 and erythropoietin on the rejuvenation of the astrocyte population. *Eur. J. Cell Biol*. **84,** 567–579.
13. Danielyan, L., Mueller, L., Proksch, B., Kabisch, D., Weller, M., Wiesinger, H., Buniatian, G.H., and Gleiter, C.H. (2005) Similar protective effects of BQ-123 and erythropoietin on survival of neural cells and generation of neurons upon hypoxic injury. *Eur. J. Cell Biol*. **84,** 907–913.
14. Shingo, T., Sorokan, S.T., Shimazaki, T., and Weiss, S. (2001) Erythropoietin regulates the in vitro and in vivo production of neuronal progenitors by mammalian forebrain neural stem cells. *J. Neurosci*. **21**, 9733–9743.
15. Studer, L., Csete, M., Lee, S.H., Kabbani, N., Walikonis, J., Wold, B., and McKay, R. (2000) Enhanced proliferation, survival, and dopaminergic differentiation of CNS precursors in lowered oxygen. *J. Neurosci*. **20**, 7377–7383.

**This article should be cited as follows:**

Danielyan, L., Buniatian, G.H., and Gleiter, C.H. (2006) Common neuroprotective effects of endothelin receptor A blockade and erythropoietin during hypoxia-associated cell injury. Perspectives of combined treatment. *TheScientificWorldJOURNAL* **6**, 449–451. DOI 10.1100/tsw.2006.89.

### BIOSKETCH

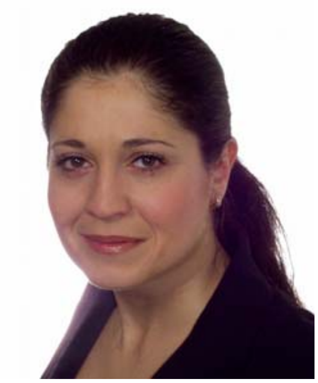

**Dr. Danielyan,** born October 5, 1970, finished Medical School in 2000 at the University of Tuebingen, Germany, and in 2001, after receiving her MD degree, she started to work in the Department of Clinical Pharmacology, University Hospital of Tuebingen. Since January 2002, Dr. Danielyan has been the leader of a group of PhD students, scientific assistants, and graduate students. The research field of her group includes the investigations on genotypic and phenotypic alterations of neural cells (neurons, astrocytes, neuronal progenitors) on hypoxic-ischemic injury, as well as Alzheimer's disease and aging. Of particular interest are investigations on modulations in the composition of cytoskeletal proteins underlying neural cell plasticity. Furthermore, she investigates the therapeutic potency of different agents influencing the local renin-angiotensin-system, endothelin-receptor-system, and erythropoietin-receptor-system in *in vitro* models of ischemia and *in vivo* pathologic conditions mentioned above.

Dr. Danielyan received 11 grants from the Medical Faculty, University of Tuebingen, pharmaceutical companies, and from private and public foundations for her scientific projects. Her results were published in *European Journal of Cell Biology* and *Biochemical Pharmacology*. In 2005, during her collaboration with Prof. W.H. Frey II, PhD (Alzheimer's Research Center, Regions Hospital, St. Paul, Minnesota), she gained an experience in intranasal delivery of substances to awake Alzheimer's transgenic mice and in the performance of cognitive tests (Morris water maze). She established the methods of quantification of gene and protein expression, of modulations of inflammatory cytokines using ELISA, as well as other new approaches for the evaluation of the efficacy of certain neuroprotectants on the cellular and molecular levels in animal models of Alzheimer's disease.
